# High schistosomiasis-related mortality in Northeast Brazil: trends and spatial patterns

**DOI:** 10.1590/0037-8682-0431-2021

**Published:** 2022-06-06

**Authors:** Bárbara Morgana da Silva, Anderson Fuentes Ferreira, José Alexandre Menezes da Silva, Rebeca Gomes de Amorim, Ana Lúcia Coutinho Domingues, Marta Cristhiany Cunha Pinheiro, Fernando Schemelzer de Moares Bezerra, Jorg Heukelbach, Alberto Novaes Ramos

**Affiliations:** 1 Universidade Federal do Ceará, Faculdade de Medicina, Pós-Graduação em Saúde Pública, Fortaleza, CE, Brasil.; 2 NHR Brasil - Nederlandse Stichting voor Leprabestrijding, Fortaleza, CE, Brasil.; 3 Universidade Federal do Ceará, Faculdade de Farmácia, Odontologia e Enfermagem, Departamento de Enfermagem, Fortaleza, CE, Brasil.; 4 Universidade Federal de Pernambuco, Centro de Ciências Médicas, Programa de Pós-Graduação em Medicina Tropical, Recife, PE, Brasil.; 5 Universidade Federal de Pernambuco, Hospital das Clínicas, Recife, PE, Brasil.; 6 Universidade Federal do Ceará, Departamento de Análises Clínicas e Toxicológicas, Laboratório de Pesquisa em Parasitologia e Biologia Molusco, Fortaleza, CE, Brasil.; 7 Universidade Federal do Ceará, Faculdade de Medicina, Departamento de Saúde Comunitária, Fortaleza, CE, Brasil.

**Keywords:** Schistosomiasis, Epidemiology, Mortality, Time series study, Brazil

## Abstract

**Background:**

We analyzed the trends and spatial patterns of schistosomiasis-related mortality in Northeast Brazil in 2000-2019.

**Methods:**

A mixed population-based ecological study was conducted, using information on the underlying or associated causes of death. We used Joinpoint regression analysis to calculate the trends. The spatial analysis included rates, spatial moving averages, and standardized mortality rates. The spatial dependence analysis was based on Getis-Ord's G and Gi* indices (Gi star) and local Moran’s index to check for autocorrelation.

**Results:**

A total of 5,814,268 deaths were recorded, of which 9,276 (0.16%) were schistosomiasis-related; 51.0% (n=4,732, adjusted rate 0.90/100,000 inhabitants [95% confidence interval (CI) 0.88-0.93]) were males; 40.0% (n=3,715, adjusted rate 7.40/100.000 inhabitants [95%CI: 7.16-7.64]) were ≥70 years old; 54.8% (n=5,087, crude rate 0.80/100,000 inhabitants) were of mixed/Pardo-Brazilian ethnicity; and 77.9% (n=7,229, adjusted rate 0.86/100,000 inhabitants [95%CI: 0.84-0.88]) lived outside state capitals. The highest proportion of deaths was in the state of Pernambuco (53.9%, n=4,996, adjusted rate 2.72/100,000 inhabitants [95%CI: 2.64-2.79]). Increasing mortality rate was verified in the state of Sergipe. On the coast of the state of Rio Grande do Norte and Bahia, there was spatial dependence of spatio-temporal risk patterns with clusters. Throughout the study period, we found positive spatial autocorrelation and cluster formation.

**Conclusions:**

In Northeast Brazil, schistosomiasis persists with a high mortality rate, especially in the coastal region, with heterogeneous spatial and temporal patterns. To eliminate schistosomiasis by 2030, it is necessary to strengthen the financing and management of the unified health system (SUS).

## INTRODUCTION

Among neglected tropical diseases (NTDs), schistosomiasis has the most severe clinical presentation and the highest number of deaths^1^ ^,^ ^2^. An estimated 232 million people are affected by schistosomiasis, and 700 million people live in endemic areas worldwide, most of them in sub-Saharan Africa^1^ ^,^ ^3^. The disease is related to precarious socio-economic conditions, lack of basic sanitation, and contamination of aquatic environments^4^ ^,^ ^5^. Schistosomiasis is included as a priority in global agendas such as the Sustainable Development Goals (SDGs) and the Roadmap for NTDs Control of the World Health Organization (WHO), which have as their main objective the elimination of the disease as a public health problem by 2030. It is envisaged that during this period, all countries will reach elimination of the disease as a public health problem, which is defined as <1% of high-intensity schistosomiasis infections^6^. Fifty-two of these 78 countries (66.7%) were considered endemic because of their moderate to high transmission intensity. In Latin America, where only intestinal schistosomiasis is recorded, Brazil, Venezuela, and the Caribbean region report most cases, and the public health relevance is evidenced by the magnitude of the number of new cases and severe clinical forms^7^ ^,^ ^8^.

In Brazil, schistosomiasis caused by *Schistosoma mansoni* is distributed mainly in nine states, seven of which are located in the northeast (Pernambuco, Sergipe, Alagoas, Bahia, Paraíba, Maranhão, and Rio Grande do Norte) and two in the southeast (Minas Gerais and Espírito Santo)^2^.

In 2017, the northeast and southeast regions of Brazil had the highest rates of positivity for schistosomiasis^9^. In the country, schistosomiasis is responsible for the second-highest burden of disability-adjusted life years among NTDs, behind Chagas disease, which is most common in the midwest, southeast, and northeast regions^10^. The northeast region of Brazil has an overall positivity rate of 6% for schistosomiasis, with higher rates in the states of Alagoas and Sergipe^12^.

According to data from the National System of Schistosomiasis Control Programme (SIS-PCE), in 2017, 91.3% of the cases, 45.7% of hospitalizations, and 64.6% of deaths were concentrated on the northeast region^4^ ^,^ ^13^. Despite the discontinuity in PCE control actions, there have been promising results in reducing the prevalence and parasite load in endemic areas^2^ ^,^ ^13^. We have seen an important reduction in the number of hospitalizations and deaths related to schistosomiasis in recent years^2^. However, new epidemiological patterns are emerging that may compromise these trends: expansion of the disease into new locations, increasing urban transmission, ecological tourism, and diagnosis difficulty in areas of low endemicity^14^ ^,^ ^15^.

In this context, systematic studies on schistosomiasis-related mortality in the northeast region of Brazil are important to identify areas with a higher frequency of the disease over time and to support public management in monitoring and evaluating the strategies and measures implemented, especially those related to the organization of health services for affected individuals. This study aimed to analyze the spatial trends and patterns of schistosomiasis mortality in the northeast region of Brazil between 2000 and 2019.

## METHODS

### Study area

**FIGURE 1:**
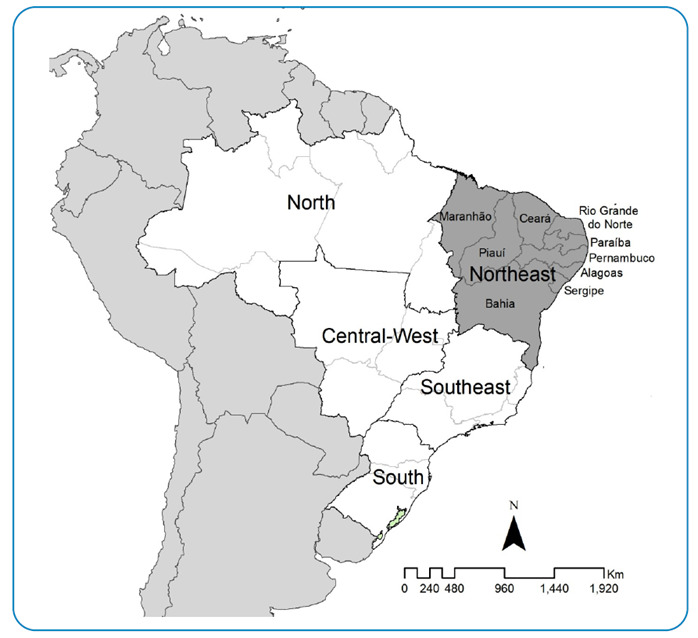
The study area includes the Northeast region of Brazil and its states.

Northeast Brazil is composed of 9 of the 27 states (Figure 1). In the country, this region has the largest number of municipalities (n=1,794/5,570; 32.2%), where approximately 28% of the Brazilian population resides. Approximately 53 million inhabitants are distributed over 1.5 million km^2^, with 48% living in rural areas^16^. It presents extensive areas with populations living under precarious socioeconomic conditions; 30.9% of the households have no access to the water supply network (approximately 12 million people without access to water on a daily basis)^17^. In this region, only 28.3% of homes have adequate sewage collection and treatment^17^.

In December 2020, the nationwide population coverage of the Family Health Strategy was 63.6%, whereas in the northeast region, the coverage was 82.3%^18^. The highest prevalence of schistosomiasis in the country is found in this region, with a high number of severe clinical forms and mortality^2^ ^,^ ^11^.

### Study design

We performed a mixed ecological study to analyze the spatial distribution and trends over time. We used information from records of schistosomiasis-related deaths (with the associated and underlying causes) in the northeast region in the International Statistical Classification of Diseases and Health Problems in its tenth revision (ICD-10). The selected causes of death were: B65-Schistosomiasis (bilharziasis); B65.0-Schistosomiasis due to *Schistosoma haematobium* (urinary schistosomiasis); B65.1-Schistosomiasis due to *Schistosoma mansoni* (intestinal schistosomiasis); B65.2-Schistosomiasis due to *Schistosoma japonicum*; B65.3-Cercarial dermatitis; B65.8-Other schistosomiases; B65.9-Schistosomiasis, unspecified. Although only *S. mansoni* is present in Brazil, we chose to include all forms to consider inappropriate classifications in death certificates (DCs).

### Data sources

Information was obtained from the Brazilian Mortality Information System (SIM). The SIM was developed by the Ministry of Health (MoH) to analyze mortality data in the country, and is based on analyzed and consolidated DCs. The DC is standardized and provided by the MoH, with free distribution throughout the national territory by the state and municipal health secretariats. It consisted of three self-copied and prenumbered copies. Medical professionals are responsible for issuing the DC, filling out all fields, and signing at the end. The DC contains the underlying and associated causes of death as well as sociodemographic and location variables related to death. For this study, we considered all deaths that mentioned schistosomiasis, either as the underlying cause or as an associated cause of death, from 2000 to 2019.

Population data were obtained from the SUS Database Department (DATASUS), based on the 2000 and 2010 Demographic Censuses and on population estimates over the intercensal period, provided by the Brazilian Institute of Geography and Statistics (IBGE) (http://tabnet.datasus.gov.br/cgi/deftohtm.exe?popsvs/cnv/popbr.def).

### Variables, statistical analysis

The following variables were analyzed: sex (male, female), age group (<15, 15-29, 30-39, 40-49, 50-59, 60-69, and ≥ 70 years), marital status (married/stable union, divorced/separated, single, and widowed), ethnicity (Caucasian, Afro-Brazilian, Asian, Mixed/Pardo-Brazilian, and Indigenous), residency in a state capital city (yes/no), state of residence (Pernambuco, Sergipe, Bahia, Alagoas, Paraiba, Rio Grande do Norte, Maranhão, Piauí, and Ceará).

Relative frequencies were calculated, including crude mortality rates (mean deaths/population) and rates adjusted for sex and age, using the direct standardization method, with the Brazilian population as a reference (2010) per 100,000 inhabitants.

The differences between groups were analyzed using the relative risk (RR) calculated with their respective confidence intervals (95%CI). We used Pearson’s chi-square (χ2) test to determine statistically significant differences between the groups.

### Trend analysis

Temporal trends in schistosomiasis-specific mortality rates were calculated using Joinpoint regression (Joinpoint Regression Program, version 4.4.2; Statistical Research and Applications Branch, National Cancer Institute, available at: https://surveillance.cancer.gov/joinpoint/). This statistical method determines the fit of a series of lines and their inflection points on a logarithmic scale using annual trend tests. Additionally, to obtain the best line for each segment, we used Monte Carlo permutation as a test of statistical significance. We tested and validated the annual percentage variation (A.P.C), with their respective 95%CIs for identification of an increase (positive A.P.C), decrease (negative A.P.C), or no trend (A.P.C without statistical significance).

### Spatial analysis

Four distinct time series were used for spatial analysis: 2000-2004, 2005-2009, 2010-2014, and 2015-2019. We included deaths in municipalities of residence in the northeast (n=1,794), excluding those with unknown municipalities of residence. The technique used to analyze the spatial distribution of deaths considered the standardized rates by age and sex, the spatial moving average (SMA), the deaths of neighboring municipalities per 100,000 inhabitants, and the standardized mortality ratio (SMR), which identifies municipalities with a number of deaths higher than expected (excess risk), dividing registered deaths by expected deaths. Thematic maps were prepared to show whether the distribution patterns remained similar and how areas with higher rates behaved over time. 

The age groups used in the calculation of mortality rates were standardized as 0-14, 15-29, 30-39, 40-49, 50-59, 60-69, and ≥70 years. The standardized rates (age and sex) were calculated by the direct standardization method using the population of Brazil in 2010 as a reference.

Spatial dependence was evaluated using the Getis-Ord indices of {G} and {Gi*} (Gi star). For this, it was assumed that a high value of score “Z” and a small value “p” of a parameter are indicative of spatial agglomeration of high values. In contrast, a low negative “Z” score and a small “p” value indicate low-value spatial clusters. These analysis parameters identified the presence of high-or low-value aggregates in groups of municipalities, providing the recognition of high-risk and low-risk clusters on the map.

The presence of local autocorrelation was verified using the local Moran’s index. The method, based on the use of local indicators of spatial association (LISA), enabled the recognition of areas with spatial associations. The results show the municipalities around which there is a significant clustering of similar values.

Statistical analyses were conducted using the Stata software (version 11.2; StataCorp LP, College Station, TX, USA), in addition to the ArcGIS software (version 9.3; Environmental Systems Research Institute, Redlands, CA, USA) for the calculation of autocorrelation indicators and building of thematic maps.

### Ethical considerations

Considering that we used a public secondary dataset without individual identification, we needed only a declaration from the Ethical Review Board of the Federal University of Ceará (Fortaleza, Brazil), exempting us from ethical clearance. The exemption from ethical clearance is backed by Law N. 12.527 of November 18, 2011, and Resolution N. 510 of 07 April 2016 of the National Health Council (CNS).

## RESULTS

### Epidemiological characteristics

A total of 9,276 schistosomiasis-related deaths were included, corresponding to 0.16% of the total deaths in the northeast region in the study period (5,814,268 deaths). The underlying cause of death in 6,681 (72.0%) DCs was identified as schistosomiasis. The average number of schistosomiasis-related deaths per year was 464. The resulting adjusted rate schistosomiasis-specific mortality rate from 2001-2019 was 0.86 (95%CI: 0.85 to 0.88) deaths per 100,000 inhabitants.

Men accounted for 51.0% (n=4,732) of deaths, with an adjusted rate of 0.90/100,000 inhabitants (95%CI: 0.88 to 0.93). The proportion of deaths was higher in the ≥70 years age group (40.0%, n=3,715), resulting in an adjusted rate of 7.40/100,000 inhabitants (95%CI: 7.16 to 7.64). Together, the age 50-59 and 60-69 years groups accounted for 42.5% of deaths, with an adjusted rate of 1.86 (95%CI: 1.77 to 1.95) and 3.91 (95%CI: 3.75 to 4.07) per 100,000 inhabitants, respectively. A total of 3,678 (39.7%) deaths were of married individuals, followed by 2,646 (28.5%) of single individuals. The predominant ethnicity was mixed/Pardo-Brazilian (54.8%, n=5,087), with a crude rate of 0.80 per 100,000 inhabitants. Non-residents of northeastern state capitals represented 77.9% (n=7,229) of the deaths, with an adjusted rate of 0.91 (95%CI: 0.87 to 0.95) per 100,000 inhabitants. Pernambuco had the highest proportion of deaths (53.9%, n=4,996), with an adjusted rate of 2.72/100,000 inhabitants (95%CI: 2.64 to 2.79) (Table 1).

**TABLE 1:** Schistosomiasis-related mortality rate according to sociodemographic variables, time trend by period based on inflection points, per 100,000 inhabitants in Northeast region, Brazil from 2000 to 2019.

Variableª	Deaths	Crude rate	Adjusted rate (95%CI)	RR^b^ (95%IC)	P-value^c^	Trends	
	No. (%)					Period	APC (95%CI)
**Total deaths**	9,276 (100.0)	0.86	0.86 (0.85 to 0.88)	-		2000-2019	-1.0^d^ (-1.6 to -0.4)
**Sexª**							
Male	4,732 (51.0)	0.89	0.90 (0.88 to 0.93)	1.09 (0.92 to 1.29)	0.3093	2000-2019	-2.0^d^ (-2.7 to -1.2)
Female	4,543 (49.0)	0.82	0.83 (0.80 to 0.85)	*1*		2000-2019	0.0 (-0.8 to 0.7)
**Age group (years)**							
< 15	34 (0.4)	0.01	0.01 (0.01 to 0.02)	0.00 (0.00 to 0.01)	<0.0001	2000-2019	-7.9^d^ (-11.7 to -3.8)
15-29	262 (2.8)	0.09	0.09 (0.08 to 0.10)	0.01 (0.01 to 0.02)	<0.0001	2000-2019	-7.3^d^ (-9.5 to -5.0)
30-39	440 (4.7)	0.27	0.28 (0.25 to 0.30)	0.04 (0.03 to 0.06)	<0.0001	2000-2019	-6.2^d^ (-8.0 to -4.4)
40-49	883 (9.5)	0.69	0.72 (0.67 to 0.76)	0.10 (0.07 to 0.13)	<0.0001	2000-2019	-7.2^d^ (-8.7 to -5.6)
50-59	1,646 (17.7)	1.86	1.86 (1.77 to 1.95)	0.26 (0.20 to 0.33)	<0.0001	2000-2019	-4.7^d^ (-5.9 to -3.6)
60-69	2,292 (24.7)	3.88	3.91 (3.75 to 4.07)	0.54 (0.43 to 0.67)	<0.0001	2000-2019	-2.6^d^ (-3.4 to -1.9)
≥ 70	3,715 (40.0)	7.21	7.40 (7.16 to 7.64)	*1*		2000-2019	-0.7 (-1.6 to 0.3)
**Marital status**							
Married/stable union	3,678 (39.7)	-	-	-	-	-	-
Divorced/separated	223 (2.4)	-	-	-	-	-	-
Single	2,646 (28.5)	-	-	-	-	-	-
Widowed	1,591 (17.2)	-	-	-	-	-	-
**Ethnicityª**							
Caucasian	2,398 (25.9)	0.77	*-*	*1*		2000-2019	-1.0^d^ (-1.8 to -0.1)
Afro-Brazilian	747 (8.1)	0.74	-	0.96 (0.69 to 1.35)	0.8318	2000-2019	-1.0 (-2.8 to 0.8)
Asian	15 (0.2)	0.12	-	0.17 (0.02 to 1.24)	0.0813	2000-2019	-18.3^d^ (-22.7 to -13.7)
Mixed/Pardo-Brazilian	5,087 (54.8)	0.80	-	1.04 (0.85 to 1.26)	0.7359	2000-2019	0.4 (-0.6 to 1.3)
Amerindian	16 (0.2)	0.38	-	0.52 (0.07 to 3.75)	0.5201	2000-2019	1.3 (-3.3 to 6.1)
**Residence in the capital**							
No	7,229 (77.9)	0.85	0.86 (0.84 to 0.88)	0.98 (0.79 to 1.22)	<0.8689	2000-2019	-3.8^d^ (-5.6 to -1.8)
Yes	2,047 (22.1)	0.87	0.91 (0.87 to 0.95)	*1*		2000-2006	2.8 (-0.2 to 6.0)
						2006-2019	-1.5^d^ (-2.3 to -0.6)
**State of residence**							
Maranhão	147 (1.6)	0.11	0.13 (0.11 to 0.15)	0.11 (0.05 to 0.23)	<0.0001	2000-2019	-1.8 (-5.1 to 1.6)
Piauí	11 (0.1)	0.02	0.02 (0.01 to 0.03)	0.03 (0.00 to 0.19)	0.0003	2000-2019	-2.1 (-6.8 to 2.9)
Ceará	121 (1.3)	0.07	0.07 (0.06 to 0.08)	0.07 (0.03 to 0.15)	<0.0001	2000-2019	-2.5 (-6.3 to 1.4)
Rio Grande do Norte	102 (1.1)	0.16	0.15 (0.12 to 0.18)	0.15 (0.06 to 0.36)	<0.0001	2000-2019	-1.2 (-4.1 to 1.7)
Paraíba	306 (3.3)	0.40	0.35 (0.31 to 0.39)	0.38 (0.21 to 0.69)	0.0016	2000-2019	-0.4 (-3.6 to 2.8)
Pernambuco	4,996 (53,9)	2.78	2.72 (2.64 to 2.79)	2.65 (1.77 to 3.96)	<0.0001	2000-2006	5.3^d^ (0.2 to 10.6)
						2006-2019	-2.7^d^ (-4.1 to -1.2)
Alagoas	1,733 (18.7)	2.71	3.08 (2.93 to 3.22)	2.59 (1.68 to 3.98)	<0.0001	2000-2006	-19.9^d^ (-34.7 to -1.7)
						2006-2019	0.2 (-6.8 to 7.8)
Sergipe	448 (4,8)	1.06	1.18 (1.07 to 1.29)	*1*		2000-2019	4,1^d^ (1,9 a 6,2)
Bahia	1,412 (15,2)	0.49	0.49 (0.46 to 0.51)	0.47 (0.30 to 0.73)	0.0008	2000-2019	2.4^d^ (1.1 to 3.8)

Missing data: ^a^ gender/sex: 1, marital status: 1,138, ethnicity: 1,013. ^b^ Relative risk; ^c^ P-value: Pearson's χ2 test; ^d^ Significantly different from 0 (P< 0.05), Monte Carlo permutation method. **APC:** annual percentage change. **CI:** confidence interval.

### Trend analysis

A more prominent negative trend in mortality was observed in men (APC -2.0; 95%CI -2.7 to -1.2). The age 15-29 years group showed the greatest downward trend in 2000-2019 (APC -7.9; 95%CI: -11.7 to -3.8), whereas the ≥70 years age group displayed no clear trend (APC -0.7; 95%CI: -1.6 to 0.3). There was a decreasing trend in deaths among people of Caucasian ethnicity (APC -1.0; 95%CI: -1.8 to -0.1), but no defined trend was observed among those of mixed/Pardo-Brazilian ethnicity (APC 0.4; 95%CI: -0.6 to 1.3). Those not residing in capital cities of the northeast region showed a decreasing mortality trend (APC -3.8 95%CI: -5.6 to -1.8) (Table 1).

Pernambuco showed an upward trend in mortality between 2000-2006 (APC 5.3; 95%CI: 0.2 to 10.6), but displayed a downward trend from to 2006-2019 (APC -2.7; 95%CI: -4.1 to -1.2). In Alagoas, there was a downward trend in the 2000-2006 period (APC-19.9; 95%CI: -34.7 to -1.7), which stabilized in the 2006-2019 period. Mortality in Sergipe showed an increasing trend during the study period (2000-2019) (APC 4.1; 95%CI: 1.9 to 6.2) (Table 1).

### Spatial analyses

**FIGURE 2:**
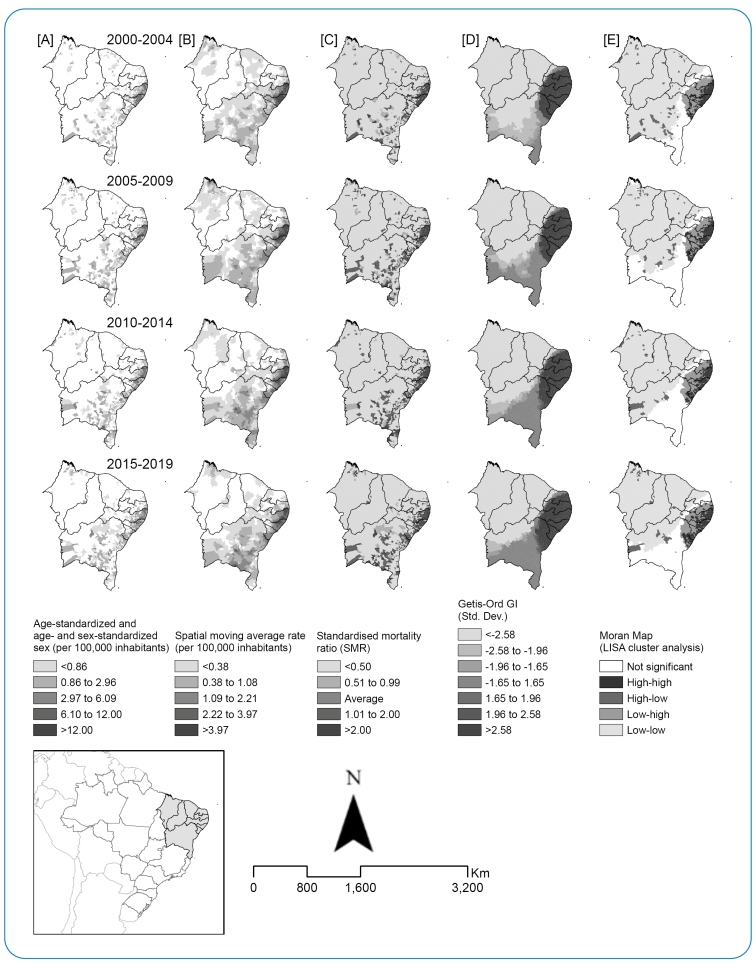
Spatial distribution of age- and sex-standardized schistosomiasis-related mortality rate **(A)**, spatial moving average rate (SMA) **(B)** (per 100,000 inhabitants), standardized mortality ratio (SMR) **(C)**, Getis-Ord GI **(D)**, and Moran's Map **(E)** according to time periods in Northeast region, Brazil from 2000 to 2019.

The spatial distribution showed a relative similarity over time, with high concentrations in specific areas. The distribution of age- and sex-adjusted mortality rates (per 100,000 inhabitants) was more pronounced in the coastal belt and the Zona da Mata states (Alagoas, Pernambuco, and Sergipe). The other states showed a lower tendency for mortality agglomeration patterns (Figure 2A). According to the SMA, high mortality rates were concentrated in Pernambuco and Alagoas throughout the study period. In 2010-2019, mortality rates increased in some areas of Bahia and Sergipe (Figure 2B). There was also an increase in the concentration of municipalities with above-average SMR in the specific areas of Pernambuco, Alagoas, Sergipe, and Bahia. However, Piauí and Ceará showed a significant reduction, particularly between 2015 and 2019 (Figure 2C).

Spatial dependence analysis using the Getis-Ord and Gi methods showed relatively similar patterns in all periods. Statistically significant spatial clustering of high values was verified in Pernambuco, Alagoas, Bahia, Sergipe, Paraíba, and Rio Grande do Norte (Figure 2D).

Local analysis of spatial autocorrelation by Moran indices identified similar positive patterns, with the occurrence of clusters in all periods (2000-2019), particularly in the coastal strip, Zona da Mata (Pernambuco and Alagoas), and southern Paraíba. However, from 2005 onwards, an increasing positive spatial autocorrelation was also observed in Sergipe and part of Bahia (Figure 2E). 

## DISCUSSION

This study reinforces the maintenance of a high schistosomiasis-related mortality burden in the main endemic areas of Brazil over a period of two decades. Significantly higher mortality rates were observed among males, those aged >50 years, those of mixed/Pardo-Brazilian ethnicity, and those who lived outside of state capital cities.

Different mortality rates in males, as well as the higher prevalence of the disease in this group, justify the importance of specific actions for this population, as indicated in previous publications^19^ ^-^ ^21^. However, the labor market and the greater prominence of women also generate occupational risks for women, which may cause changes in the mortality patterns in the future^19^. Other studies found that socioeconomic and occupational indicators were more relevant for estimating the risk of infection and death from schistosomiasis than gender-focused assessment^9^ ^,^ ^22^ ^,^ ^23^.

The maintenance of lower mortality rates in younger people (under 15 years) may be related to this population being the target of systematic control measures implemented by the PCE, which are based primarily on WHO recommendations. During the 1980s and the 1990s, the PCE instituted in Brazilian states and municipalities undertook routine fecal surveys to estimate the prevalence and performed mass treatment in endemic areas^2^ ^,^ ^9^ ^,^ ^22^ ^,^ ^23^. These surveys usually consisted of sampling schoolchildren aged 7-14 years, followed by mass drug administration (MDA) when the prevalence was higher than 50% in this population. In many situations, sample-based surveys did not recognize the minimum prevalence levels required for mass treatment, and selective individual treatment of cases was carried out instead^2^ ^,^ ^9^ ^,^ ^22^ ^,^ ^23^. The adoption of this strategy may have reduced infection in young people and, consequently, severe disease and deaths in this age group. It is possible that some of these children migrated to other regions of Brazil or to urban areas of the same state or municipality, where they had no contact with *S. mansoni*. In contrast, those who persisted in areas of higher endemicity may have reinfected themselves and were more likely to develop more severe clinical forms, with potential progression to death^21^ ^,^ ^22^.

This may also explain the maintenance of higher mortality rates in older people. In addition to being a chronic condition, the higher mortality risk in the population aged ≥ 70 years may indicate either continuous exposure to infection, low quality of life, or even the coexistence of other infectious or non-infectious diseases^9^ ^,^ ^11^ ^,^ ^23^. The coexistence of schistosomiasis with other age-specific physiological conditions caused a tendency towards greater vulnerability in this age group^23^ ^-^ ^25^.

More than half of the deaths associated with schistosomiasis in the northeast were concentrated in Pernambuco. This finding highlights the relevance of endemic disease to the state and corroborates other studies^19^ ^,^ ^24^. In 2011, Pernambuco had the third-highest prevalence of schistosomiasis among the northeast states, which occurred in 93 (50.0%) municipalities, with an annual average of 180 deaths (from 2005 to 2014)^26^ ^,^ ^27^. Other states in the northeast regions of Brazil (Pernambuco, Alagoas, Sergipe, and Bahia) have shown higher mortality burdens, especially in areas with high positivity, as verified by surveys conducted during in 2010-2015^9^.

The higher proportion of deaths among people of mixed/Pardo-Brazilian ethnicity, with higher relative risk, corroborates the other findings, which possibly points to the existence of critical ethnic and social inequalities in Brazil^20^ ^,^ ^28^. The persistence of social determinants favoring infection and disease severity may contribute to the deaths^29^. However, several studies on mortality related to the disease have shown that the mixed/Pardo-Brazilian ethnicity is not directly associated with a higher risk, but it is more important than other indicators as a proxy of socioeconomic condition^20^ ^,^ ^30^.

The present study showed a higher frequency of deaths in residents outside the capital, which corroborates previous findings that schistosomiasis still has rural characteristics, with transmission predominating in areas where there are water collections and populations that, for sociocultural and economic reasons, develop their domestic, occupational, personal hygiene, and leisure activities in these environments. Environmental factors associated with the lack of water supply and treatment for human consumption, inadequate sanitation, and intense migration create conditions conducive to the maintenance of transmission and the continued expansion of the endemic disease. In addition, there are difficulties in accessing health centers in these areas^20^.

Schistosomiasis as an associated cause of mortality was reported in almost 30% of DCs. An analysis based exclusively on the underlying cause of death would underestimate the actual mortality burden of the disease. Analysis of multiple causes of death increases sensitivity, minimizing possible failures resulting from registration^20^ ^,^ ^24^. The inability of health services to identify and treat schistosomiasis in a timely manner, which is associated with underreporting of deaths, may contribute to the high and persistent mortality burden, especially in regions with higher endemicity^19^ ^,^ ^24^.

The higher concentration of deaths distributed along the coastal belt and in the area of the Zona da Mata, especially in Alagoas and Pernambuco, as verified by other studies, indicates the sustained transmission of the disease in endemic areas located in the interior of the states, and spatial analyses of mortality have also recognized a higher risk for death due to the disease in these areas^9^ ^,^ ^13^ ^,^ ^24^ ^,^ ^31^ ^-^ ^35^.

The high clustering of deaths and strong positive spatial autocorrelation in historically endemic areas (such as Pernambuco and Alagoas), and more recently in some areas of Sergipe and Bahia, indicates its expansion through the formation of new clusters, especially in 2015-2019^13^ ^,^ ^19^ ^,^ ^20^ ^,^ ^31^ ^,^ ^36^.

The limitations of this study were particularly due to the use of secondary data. Death records may have been incomplete and inconsistent (for example, owing to the significant number of deaths coded as unspecified schistosomiasis; data not presented). We also recognize the difficulty of the health system in characterizing schistosomiasis as an underlying cause or even as an associated cause of death. For example, cases with the severe hepatosplenic form, which is associated with alcoholism, may compromise the registration of the disease as an underlying cause, making classic epidemiological analysis of the disease difficult. Some deceased may not have been submitted to necropsy evaluation in Death Verification Services, making it impossible to consistently define the underlying cause of death.

We also point out that in endemic areas of the disease, cases are registered in the Schistosomiasis Control Program Information System (SISPCE), which, although it has some limitations because it is used only in fecal surveys and without individual identification of positive cases, is still the official system for carrying out epidemiological analyses and defining strategies for the surveillance and control of the disease.

The programmatic vulnerability of this population, characterized by limited access to reference health services for disease management, may also have contributed to this limitation.

We conclude that, despite the general trend of decreasing schistosomiasis mortality in Northeast Brazil, there was a persistence of high levels, with heterogeneous spatial and temporal patterns over the 20 years analyzed. In recent years, some states (Sergipe and Bahia) have shown an increasing trend in disease-related mortality rates. The coastal strip of the northeast region still has high mortality rates, reaching urbanized populations in this region. Our findings indicate the permanence of the transmission cycle due to deficiencies in basic sanitation, low access to health centers, and delays in diagnosis and treatment. The high risk of death in individuals indicates the need for strategic control measures focused on specific target populations, such as men, the elderly, and residents outside the state capital. The high mortality burden in the northeast region calls for systematic epidemiological studies using spatial and temporal approaches to provide elements for planning surveillance and control activities. Strengthening the financing and management of Brazil’s unified health system, SUS, is paramount to reach the elimination goal by 2030.
